# Characterization of the intracellular neurexin interactome by *in vivo* proximity ligation suggests its involvement in presynaptic actin assembly

**DOI:** 10.1371/journal.pbio.3002466

**Published:** 2024-01-22

**Authors:** Marcos Schaan Profes, Araven Tiroumalechetty, Neel Patel, Stephanie S. Lauar, Simone Sidoli, Peri T. Kurshan

**Affiliations:** 1 Department of Neuroscience, Albert Einstein College of Medicine, Bronx, New York, United States of America; 2 Department of Biochemistry, Albert Einstein College of Medicine, Bronx, New York, United States of America; Columbia University Medical Center, UNITED STATES

## Abstract

Neurexins are highly spliced transmembrane cell adhesion molecules that bind an array of partners via their extracellular domains. However, much less is known about the signaling pathways downstream of neurexin’s largely invariant intracellular domain (ICD). *Caenorhabditis elegans* contains a single neurexin gene that we have previously shown is required for presynaptic assembly and stabilization. To gain insight into the signaling pathways mediating neurexin’s presynaptic functions, we employed a proximity ligation method, endogenously tagging neurexin’s intracellular domain with the promiscuous biotin ligase TurboID, allowing us to isolate adjacent biotinylated proteins by streptavidin pull-down and mass spectrometry. We compared our experimental strain to a control strain in which neurexin, endogenously tagged with TurboID, was dispersed from presynaptic active zones by the deletion of its C-terminal PDZ-binding motif. Selection of this control strain, which differs from the experimental strain only in its synaptic localization, was critical to identifying interactions specifically occurring at synapses. Using this approach, we identified both known and novel intracellular interactors of neurexin, including active zone scaffolds, actin-binding proteins (including almost every member of the Arp2/3 complex), signaling molecules, and mediators of RNA trafficking, protein synthesis and degradation, among others. Characterization of mutants for candidate neurexin interactors revealed that they recapitulate aspects of the *nrx-1(-)* mutant phenotype, suggesting they may be involved in neurexin signaling. Finally, to investigate a possible role for neurexin in local actin assembly, we endogenously tagged its intracellular domain with actin depolymerizing and sequestering peptides (DeActs) and found that this led to defects in active zone assembly. Together, these results suggest neurexin’s intracellular domain may be involved in presynaptic actin-assembly, and furthermore highlight a novel approach to achieving high specificity for *in vivo* proteomics experiments.

## Introduction

The proper formation of synaptic connections underlies our brain’s ability to form appropriate neuronal circuits, and defects in this process lead to neurodevelopmental and neuropsychiatric disorders. Synaptic cell-adhesion molecules (sCAMS) are thought to play a role in both the specificity of this process, by selecting appropriate synaptic partners [1–3], and in the stabilization and functional maturation of nascent synapses [4,5].

Neurexins constitute a family of presynaptic CAMs that are highly associated with autism and schizophrenia [6], and are thought to function as central “hubs” of *trans*-synaptic interaction [7]. The synaptogenic activity of neurexin was initially demonstrated by showing that binding to its canonical binding partner neuroligin could induce the formation of hemi-presynapses in cultured neurons [8–10]. The human genome encodes 3 neurexin genes, which together can be expressed as approximately 4,000 different splice isoforms [11,12]. These isoforms contain a mostly invariant intracellular domain (ICD) responsible for a largely uncharacterized downstream intracellular signaling pathway: the intracellular C-terminal PDZ-binding motif (PBM) of neurexin interacts with the synaptic vesicle (SV) protein synaptotagmin as well as the scaffolding proteins Cask and Mint [13–16]. In addition, *Drosophila* neurexin has been shown to interact with the active zone (AZ) protein dSYD-1 [17] as well as the actin-binding protein spinophilin [18].

*Caenorhabditis elegans* contains a single neurexin gene (nrx-1) that encodes both long and short isoforms [19,20]. The long isoforms of NRX-1 have been implicated in neurite outgrowth, synapse specificity, and postsynaptic organization [21,22], while the short isoform is sufficient for presynaptic maturation and stability [20]. Using markers for presynaptic assembly including the SV-associated protein RAB-3 and the AZ protein clarinet (CLA-1; homolog of vertebrate AZ protein Piccolo [23]), we have previously shown that *C*. *elegans* NRX-1 stabilizes nascent synapses and is required for their morphological and functional maturation [20]. However, the downstream signaling pathways responsible for these functions remain unknown.

To better understand the molecules that might mediate neurexin’s presynaptic role in synapse stabilization and maturation, we have employed the enzyme-catalyzed proximity-labeling approach TurboID [24]. This method utilizes the promiscuous biotin ligase BirA, fused to a protein of interest, to allow for biotinylation of target proteins within a radius of a few nanometers. Biotinylated proteins are pulled down with streptavidin and identified by mass spectrometry. Unlike traditional biochemical approaches, this method does not require interacting proteins to remain in complex during purification, a particular advantage when studying transmembrane proteins or looking for transient interactions. While proximity ligation methods have been extensively validated in cultured cells, their application *in vivo* has only recently begun to reveal important biological interactions [25,26].

To identify proteins that interact with neurexin intracellularly, we used CRISPR gene editing to endogenously tag the neurexin intracellular domain with TurboID and confirmed that this does not affect neurexin function *in vivo*. Streptavidin pull-downs and mass spectrometry were used to identify biotinylated proteins. We then compared our results to 3 different negative controls: a wild-type strain (N2 Bristol) lacking any TurboID protein, a strain over-expressing cytosolic TurboID pan-neuronally, and a strain in which TurboID was endogenously tagged to NRX-1, but in which the PBM of NRX-1 had been deleted leading to a de-clustering of NRX-1 from presynaptic active zones. This “ΔPBM” negative control is thus expressed from the endogenous locus, and thus in the same cells and likely at the same levels as the experimental strain and differs only in its specific localization at synapses. By comparing our experimental strain with the 3 different control strains, we find that the ΔPBM strain is the most appropriate negative control, the former 2 being too permissive or too restrictive, respectively. Using this control, we have generated a list of potential NRX-1 interactors, including both known and novel binding partners. These include presynaptic active zone proteins as well as many proteins involved in remodeling of the actin cytoskeleton. We characterized mutants for a subset of these proteins and discovered that they recapitulate aspects of the *nrx-1(-)* mutant phenotype, suggesting they may be involved in neurexin signaling. Finally, to directly assess the role of actin polymerization in neurexin’s presynaptic function, we fused a bacterially derived actin-sequestering peptide Gelsolin1 (GS1) to neurexin’s ICD and found that this resulted in a pronounced reduction in active zone size.

## Results

### Endogenous tagging and validation of neurexin with intracellular TurboID

Neurexin mutants have a defect in presynaptic assembly and stability and thus are more susceptible to extrinsic inhibitory cues, the result of which is that they have fewer active zones clusters, particularly at the edges of the synaptic domain (where inhibitory cues are highest) [20]. They also exhibit an increase in the number of small, highly mobile SV precursor packets in the asynaptic region of the axon [20]. These dual phenotypes allow us to assess neurexin function using a transgenic marker that expresses both a fluorescently tagged active zone protein (Clarinet, or CLA-1 [23]) and an SV protein (RAB-3) in the DA9 motor neuron in the tail of the worm [20].

**Fig 1:**
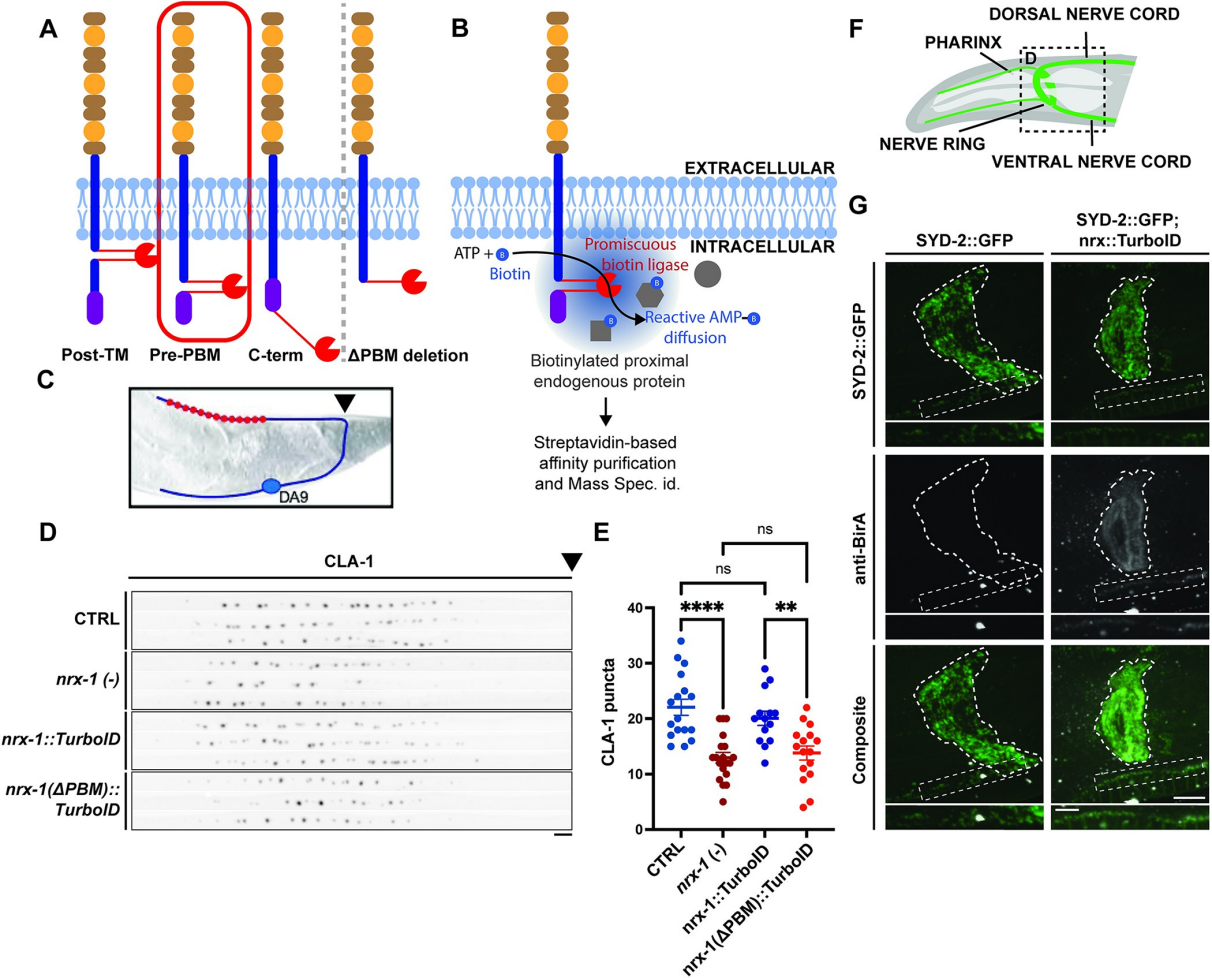
Generation and validation of an endogenously tagged neurexin-TurboID strain and control. (**A**) Left: Schematic depicting several insertion sites of TurboID enzyme that were assessed, with the final chosen and validated site circled in red. Right: Schematic of the neurexin-ΔPBM-TurboID control strain. (**B**) Schematic of the rationale and workflow for the proteomics screen. (**C**) Schematic of the DA9 motor neuron used to assess presynaptic assembly phenotypes. Arrowhead points to where cropped images begin in D. (**D**) Straightened images of CLA-1-GFP puncta in the DA9 synaptic domain across different genotypes. Scale bar: 4 μm. (**E**) Quantification of CLA-1 puncta number in the indicated genotypes reveals that our experimental strain (neurexin-TurboID) does not impact neurexin function, but our ΔPBM negative control strain does. (**F**) Schematic of the synapse-rich nerve ring in the head of the worm. (**G**) Immunohistochemistry using anti-BirA antibody compared to GFP fluorescence of endogenously tagged active zone protein SYD-2 in the nerve ring and nerve cord (insets) reveals synaptic localization of our endogenously tagged neurexin-TurboID. Scale bars: 10 μm for nerve ring images and 5 μm for insets. PBM, PDZ-binding motif.

The ICD of neurexin is largely uncharacterized and contains few sequence motifs, with the notable exception of a C-terminal PBM. To identify an appropriate location within neurexin’s ICD in which to insert the TurboID biotinylating enzyme (BirA), we considered 3 options: (1) just after the transmembrane domain; (2) just before the PBM; and (3) at the very C-terminus with an extra-long linker (Fig 1A). We generated rescue constructs of each and assayed their ability to rescue the neurexin null (*nrx-1(-)*) phenotype, using the marker described above. Insertions at the first 2 locations were able to rescue the null phenotype; however, the third (C-terminal) option failed in rescuing the phenotype, thus it was discarded. We proceeded to generate TurboID endogenous CRISPR knock-in strains of the endogenous neurexin locus at the other 2 ICD locations (see Materials and methods). In contrast to our over-expression rescue experiments, the first (post-transmembrane domain) led to a *nrx-1(-)* phenotype, indicating that the endogenous insertion had abrogated neurexin’s function. However, the second (pre-PBM; Fig 1A and 1B), resulted in wild-type presynaptic development (Fig 1C–1E), suggesting that the insertion of TurboID at this location did not impact neurexin function in presynaptic assembly and stability.

We further validated this strain by performing immunocytochemistry on our TurboID-tagged neurexin strain, using antibodies against BirA, and comparing the pattern of expression to another endogenously tagged presynaptic active zone protein, SYD-2/Liprin-α [27]. Expression of both neurexin-TurboID and SYD-2-GFP colocalized well in the synapse-rich region of the nerve ring (Fig 1F and 1G), as well as in the individual puncta of the nerve cord (Fig 1G, insets), indicating that neurexin-TurboID was localizing appropriately to presynaptic active zones.

Previous TurboID experiments in *C*. *elegans* have made use of a negative control strain in which cytosolic BirA is over-expressed in the tissue of interest through the use of an integrated multi-copy array [26]. To generate a more appropriate and highly specific negative control strain for our TurboID proteomics experiments, we genetically removed the PBM from our endogenously tagged neurexin-TurboID strain (see Materials and methods and Fig 1A), as this leads to the de-clustering of neurexin and its dispersal along the cell surface [28]. Indeed, the deletion of the PBM in the neurexin-TurboID strain led to a synaptic assembly phenotype similar to that of the *nrx-1(-)* mutant with a decrease in the number of active zones labeled by CLA-1 (Fig 1D and 1E) as well as an increase in small, asynaptic vesicle precursors labeled by RAB-3 (S3B Fig), indicating that neurexin’s localization at active zones is critical to its function in presynaptic assembly and stability.

### Proteomics results and comparison to multiple negative control strains

**Fig 2:**
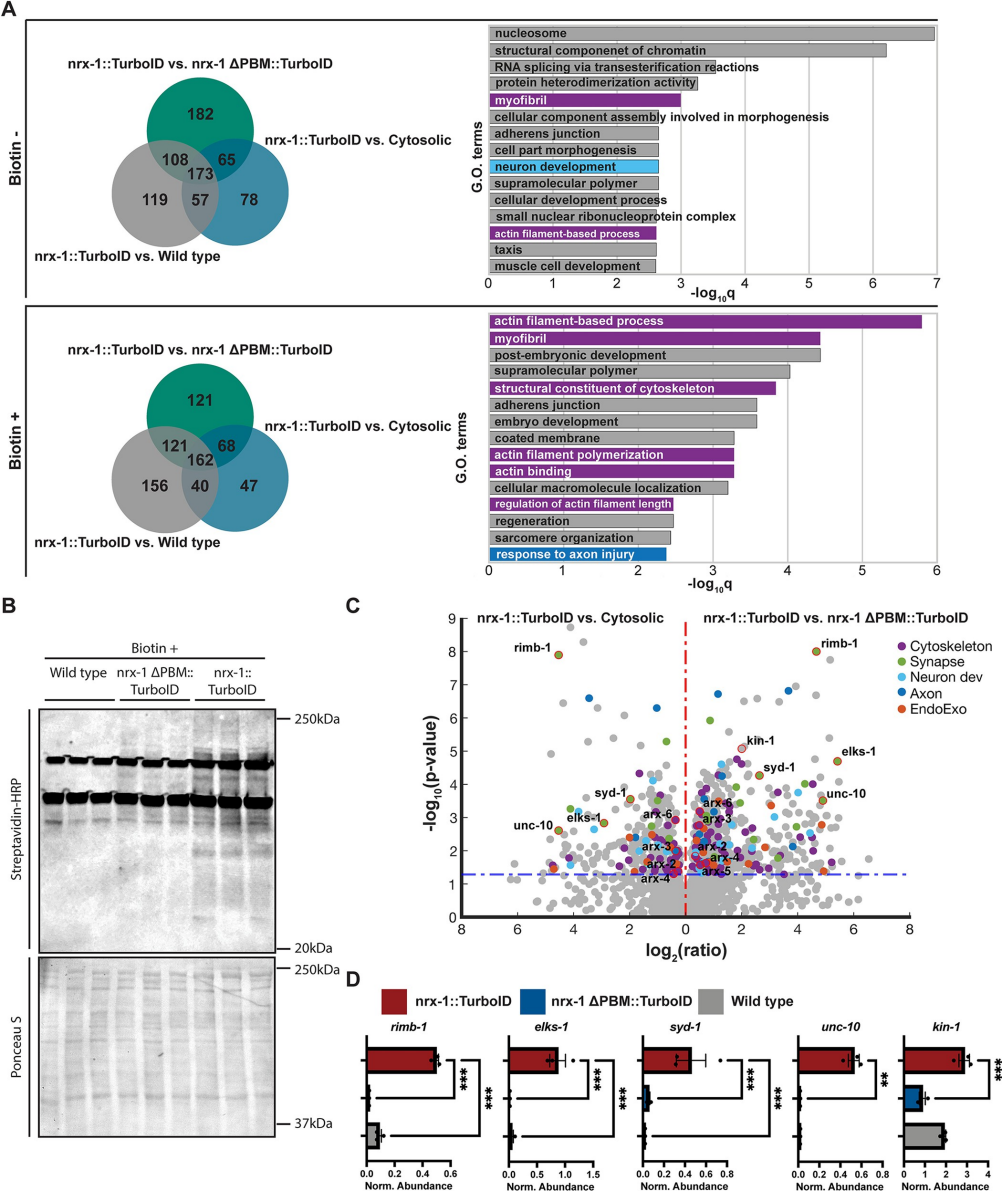
Comparison of proteomics results between multiple negative control strains. (**A**) Left: Venn diagrams showing proteins enriched in our experimental strain (neurexin-TurboID) compared to 3 different negative control strains (wild-type N2, pan-neuronal cytosolic TurboID, and neurexin-ΔPBM-TurboID), in both basal and enriched Biotin conditions. Right: GO terms of most highly enriched genes in comparison to neurexin-ΔPBM-TurboID in both basal and enriched biotin conditions. (**B**) Western blot of biotinylated proteins in our experimental strain (neurexin-TurboID, right columns) compared to 2 controls (wild type, left columns and neurexin-ΔPBM-TurboID, middle columns) in the enriched biotin condition as probed by streptavidin-HRP. Total protein levels (as assessed by Ponceau staining, lower blot) were used as a loading control. (**C**) Volcano plot of genes corresponding to the proteins enriched in our experimental strain (neurexin-TurboID) compared to 2 negative controls (enriched in comparison to over-expressed cytosolic pan-neuronal TurboID on the left and enriched in comparison to neurexin-ΔPBM-TurboID on the right). (**D**) Normalized abundance compared to 2 controls (wild type and neurexin-ΔPBM-TurboID) for known active zone components likely to be closely physically associated with neurexin’s intracellular domain using data from S2 Table. GO, Gene Ontology; PBM, PDZ-binding motif.

To identify candidate proteins that may interact with neurexin’s intracellular domain, we set out to perform proteomics analysis of our endogenous neurexin-TurboID strain, compared to 3 different negative control strains: wild type (N2), which contains no BirA enzyme; the pan-neuronally over-expressed cytosolic TurboID strain (wyIs867); and our newly generated neurexin-ΔPBM-TurboID strain (Fig 2). Six replicates of developmentally synchronized worms enriched for adults were grown on standard bacterial medium (OP50, which contains low levels of biotin). Two hours prior to their lysis, half (3) of the replicates of each strain were incubated on media supplemented with an additional 1 mM of biotin based on previously optimized conditions [25].

The lysates from each strain/condition were then used to perform streptavidin pull-downs to isolate biotinylated proteins (see Materials and methods and Fig 1B). Following pull-downs, we performed western blots to assess and validate our purification and to control for BirA protein biotinylation (Figs 2B and S1A). Total protein levels (as assessed by Ponceau staining; Figs 2B and S1A) were used as a loading control and biotinylated proteins were assessed by immunoblotting with streptavidin-HRP. The experimental strain, neurexin-TurboID, showed increased biotinylated protein levels and resulted in more easily identified specific bands following streptavidin immunoblotting (Fig 2B) when compared to the controls. This was particularly noticeable in the added-biotin conditions (compare Fig 2B to S1A Fig), suggesting an increase in specificity in this condition. Following streptavidin pull-downs, samples were analyzed by mass spectrometry. When comparing the biotin-enriched condition to the basal condition, we saw an increase in the number of candidate genes with Gene Ontology (GO) terms predicted to be relevant to neurexin function (e.g., synapse, neuron development, axon, endo/exocytic-related, and cytoskeleton; S1B Fig). Additionally, these hits displayed higher fold-change and/or *p*-value compared to the non-biotin-enriched samples, again suggesting increased specificity in the biotin-enriched condition.

We confirmed that the different samples clustered according to their strain and biotin condition using PCA and k-means clustering (S2 Fig). We carried out an ANOVA test to identify proteins enriched in our experimental strain (neurexin-TurboID) relative to the 3 negative control strains (wild type, cytosolic TurboID, and neurexin-ΔPBM-TurboID) in both biotin conditions, and constructed Venn diagrams of the overlapping hits using a 95% confidence threshold for including candidates (Fig 2A).

Using a combination of GO (Fig 2A) and volcano plots (Fig 2C) to compare these enriched hits in our experimental strain relative to either the overexpressed cytosolic TurboID or our neurexin-ΔPBM-TurboID controls, we found a greater number and enrichment of relevant neuronal, synaptic, and cytoskeletal terms in the latter condition. This was particularly true for the biotin-enriched samples, where these hits were both further enriched and higher up on the GO list (Fig 2A). We interpreted this as indicating that the over-expressed cytosolic TurboID, due to its high expression level, may obscure real neurexin interactors. This might especially be the case for interactors that are themselves highly expressed throughout the cell, such as cytoskeletal proteins, thus making this strain too stringent a negative control. For example, GO analysis of candidate interactors obtained using the neurexin-ΔPBM-TurboID as a control revealed an increase in actin-related terms as compared with using the cytosolic TurboID control (Fig 2C). Indeed, out of the 40 actin-related enriched proteins found using the neurexin-ΔPBM-TurboID control, 15 would have been lost if the neuronal cytosolic TurboID were used instead.

Tissue enrichment analysis (TEA) as well as identification of several components of the presynaptic active zone, including RIMB-1, ELKS-1, SYD-1, and UNC-10/Rim (Figs 2D and S1C), gave us confidence in the specificity of our results. Importantly, the *Drosophila* orthologs of neurexin and SYD-1 have been found to be direct binding partners [17]. We also found enrichment of the *C*. *elegans* PKA ortholog KIN-1 (Fig 2D), the mammalian version of which has been implicated in regulating presynaptic potentiation downstream of neurexin [29]. Interestingly, very few of the actin-related proteins enriched in our screen were also enriched in a previous screen for ELKS-1 interactors [25] (S1D Fig), suggesting that enrichment of actin-related proteins is not simply a nonspecific outcome of tagging a presynaptic protein. Overall, we concluded that our specific endogenous control strain (neurexin-ΔPBM-TurboID) is the most appropriate control strain, since it is expressed from the endogenous locus and promoter (and therefore likely at similar levels to our experimental strain) and differs only in its subcellular localization pattern (loss of synaptic enrichment [28]), and we proceeded in our analysis using that comparison.

### Neurexin interactions with novel proteins and signaling pathways

**Fig 3:**
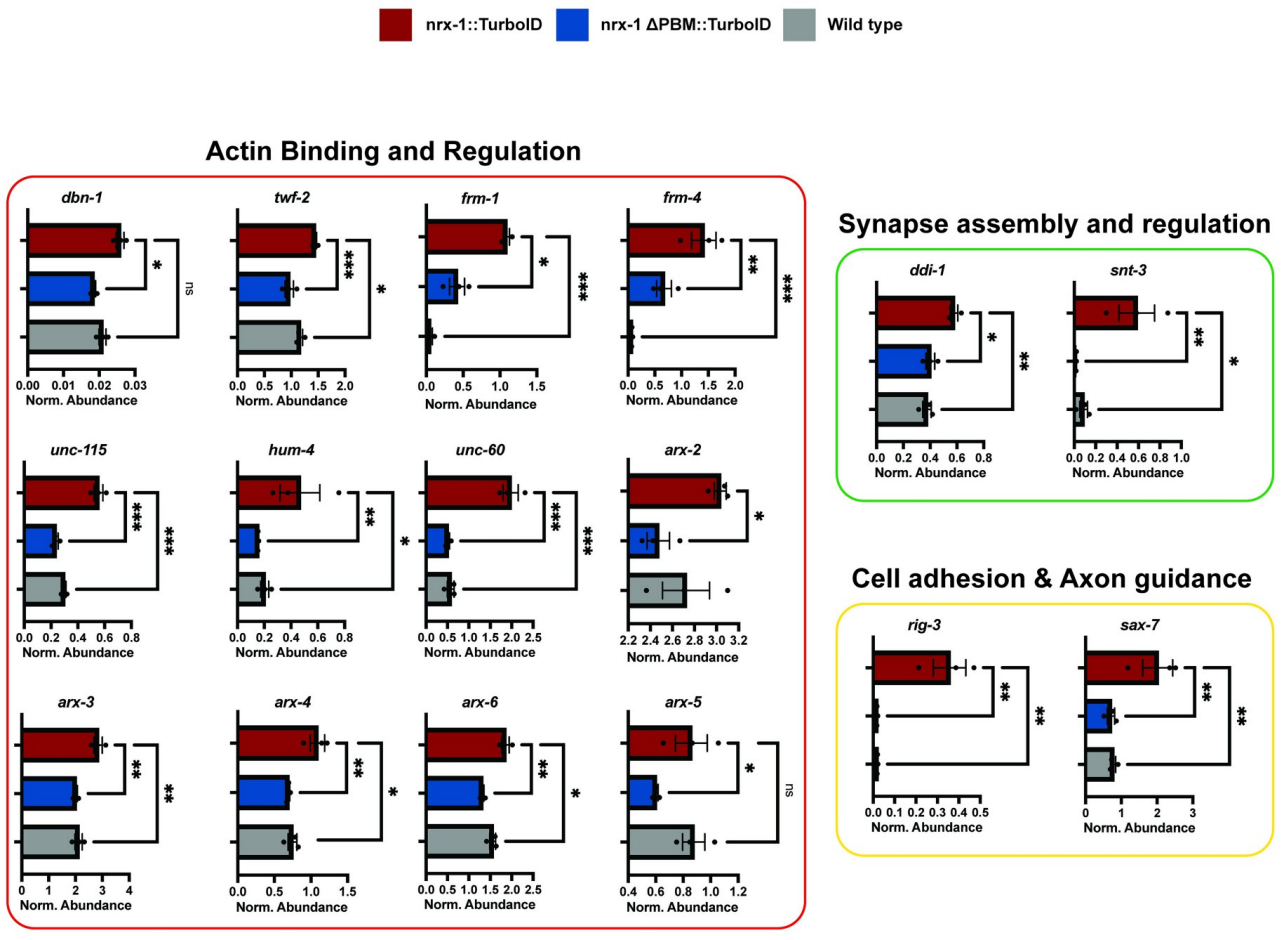
Candidate neurexin interactors in multiple molecular pathways. Normalized abundance compared to 2 controls (wild type and neurexin-ΔPBM-TurboID) for a subset of genes of interest ordered by their GO terms using data from S2 Table. GO, Gene Ontology.

Having determined the most appropriate negative control, we began our analysis of candidate interacting proteins revealed by the proteomics analysis. To select those, we used a 95% pairwise confidence threshold for including candidates. We found candidate interactors that fell into several broad classes: active zone proteins (Fig 2D), cytoskeletal-associated proteins, in particular actin-related proteins, including most members of the actin-nucleating Arp2/3 complex (*arx* genes in *C*. *elegans*), other actin-associated proteins (FRM-1, FRM-4, HUM-4, DBN-1, TWF-2, UNC-115, and UNC-60), additional synaptic proteins (DDI-1, SAX-7), as well as those involved other neuronal processes (Fig 3). We plotted the normalized abundance for each protein in each condition (experimental, ΔPBM, and wild-type strains) for easier comparison (Fig 3).

### Mutants of candidates from proteomics screen partially phenocopy neurexin mutants and have varied effects on synapse assembly/stability

**Fig 4:**
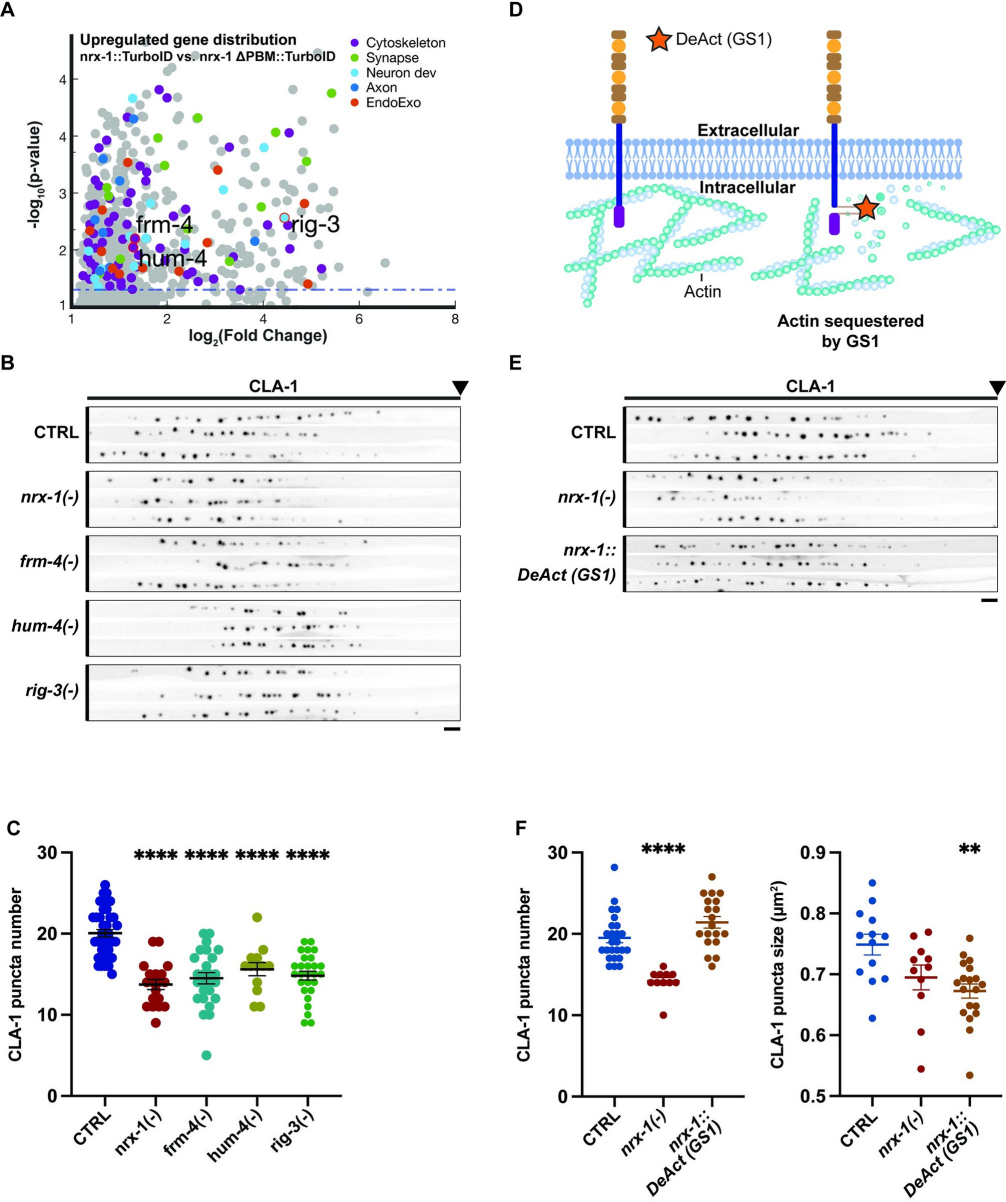
Validation of actin-binding proteins by mutant analysis and DeAct tagging. (A) Zoom in of Semi-Volcano plot of genes corresponding to the proteins enriched in our experimental strain (neurexin-TurboID) compared to control (neurexin-DPBM-TurboID), replotted from Fig 2C, but with selected candidate interactor genes highlighted to show their relative enrichment within the dataset. (B) Straightened images of CLA-1-GFP puncta in the DA9 synaptic domain across different genotypes. Scale bar: 4 μm. (C) Quantification of CLA-1 puncta number in the indicated genotypes. (D) Schematic depicting the insertion site of DeAct tool GS1. (E) Straightened images of CLA-1-GFP puncta in the DA9 synaptic domain across wild type, *nrx-1(-)* and *nrx-1*::*DeAct(GS1)* genotypes. Scale bar: 4 μm. (F) Quantification of CLA-1 puncta number and size in the indicated genotypes in E. GS1, Gelsolin segment 1.

We focused our attention on several candidate interactors that, while not previously associated with neurexin, were predicted to be involved in cytoskeletal or cell adhesion-related pathways. Null mutants for these genes, generated by the *C*. *elegans* Deletion Mutant Consortium [30], were obtained from stock centers (see strain list in Materials and methods) and crossed to our synaptic marker strain and assessed for presynaptic assembly defects. These include *frm-4*, *hum-4*, and *rig-3* (Fig 4A). *Frm-4* encodes a FERM domain-containing protein predicted to be involved in actomyosin structure organization, *hum-4* (heavy chain of an unconventional myosin) encodes a protein that is predicted to enable actin filament binding activity and *rig-3* (neuRonal IGCAM) encodes an adhesion molecule located in axons and synapses.

Compared to wild-type animals, *nrx-1(-)* mutants exhibit an approximately 30% reduction in the number of active zones (CLA-1 puncta), primarily within the proximal synaptic domain, as well as an increase in small, asynaptic vesicle precursors (RAB-3 puncta) in the axon commissure (Figs 4B, 4C and S3, and [20]). The *frm-4(-)*, *rig-3(-)*, and *hum-4(-)* mutants all showed a pronounced reduction in CLA-1 puncta in comparison to wild type (Fig 4B and 4C). The *frm-4(-)* and *hum-4(-)* mutants also recapitulated the increase in asynaptic RAB-3 seen in the *nrx-1(-)* mutant (S3B Fig). The fact that disparate candidate interactors seem to regulate distinct aspects of neurexin function suggests that neurexin may function upstream of several different pathways controlling presynaptic assembly and stability.

### Neurexin’s intracellular domain may regulate presynaptic actin organization and/or polymerization

Our GO analysis showed a prominent enrichment in actin-related proteins including almost all the members of the actin-nucleating Arp2/3 complex (arx genes in *C*. *elegans*) and other actin-associated proteins (FRM-1 FRM-4, HUM-4, DBN-1, TWF-2, UNC-115, and UNC-60; Figs 2, 3, and S4). Additionally, only 8 of the 40 genes with actin-related function overlapped with hits from a previous TurboID screen of the endogenously tagged active zone protein ELKS-1 [25], demonstrating the specificity of these interactions (S1D Fig). Due to the importance of the actin cytoskeleton in presynaptic structure and organization, redundant signaling pathways are likely involved, making single-mutant analysis hard to interpret. Moreover, many actin proteins are essential in worms and their mutants therefore lethal. To understand whether neurexin may mediate very local changes in actin organization, we decided to employ a strategy aimed at specifically perturbing actin polymerization surrounding neurexin’s intracellular domain. DeActs are a class of bacterially derived, genetically encoded actin-modifying polypeptides, that can induce actin disassembly in eukaryotic cells [31]. Using CRISPR/Cas9, we endogenously tagged neurexin’s ICD with the DeAct Gelsolin segment 1 (GS1), a ∼120-amino-acid domain that sequesters actin monomers, placing it in the same location that we had previously inserted TurboID (Fig 4D). We found that in neurexin-GS1, the number of active zones marked by the active zone scaffold Clarinet (CLA-1) was unaltered; however, the average size of CLA-1 puncta was decreased (Fig 4E and 4F), a defect in active zone assembly even more pronounced than that found in neurexin mutants. Altogether, our data suggest that neurexin may mediate presynaptic assembly in part by interacting with factors regulating actin polymerization and/or organization.

## Discussion

Here, we report the use of *in vivo* proximity labeling to identify intracellular interactors of the synaptic cell-adhesion molecule neurexin. We have targeted neurexin’s intracellular domain, a region common to all neurexin genes and isoforms and thus critical for mediating neurexin signaling in all neuronal contexts. Moreover, we have conducted this analysis using endogenously tagged neurexin *in vivo*, thus retaining the appropriate cellular context and abrogating any effects of over-expression. Careful selection and validation of the endogenous insertion site resulted in generation of an experimental strain with wild-type neurexin function, while an analysis of several possible negative control strains led to the selection of the most appropriate one. Indeed, our control strain is not only well-matched for expression levels, but also differs from the experimental strain only in its synaptic localization, thus removing interactors from other subcellular domains such as within the secretory pathway. We have identified both known and novel candidate interactors of neurexin’s intracellular domain, revealing unknown roles for these proteins in presynaptic assembly and stability. In particular, we have identified a likely role for neurexin in actin nucleation, due to the identification of almost every member of the actin-nucleating Arp2/3 complex in our proteomic screen results (an enrichment that was not found for other TurboID experiments conducted on *C*. *elegans* active zone proteins [25]).

### Actin regulation may underlie neurexin’s role in presynaptic assembly

Arp2/3 is crucial for regulation of both the initiation of actin polymerization and organization of the resulting filaments into branched networks [32]. Actin polymerization has been shown to be required for the development of synaptic structures and the clustering of SVs within presynaptic boutons [33]. In *Drosophila*, neurexin has been shown to interact genetically with the actin-binding protein spinophilin [18]. However, a direct connection between neurexin signaling and actin polymerization has not yet been reported. Although more studies are required to validate a direct link between neurexin and actin polymerization, the enrichment of actin-binding and actin-nucleating proteins in our proteomics results (and lack of one reported in other active zone proteomics experiments [34]), coupled with the pronounced effect on active zone size obtained by fusing the DeAct peptide GS1 to neurexin’s intracellular domain, suggests that neurexin may play a role in actin modification at the active zone, although the effects of the DeAct peptide may also be nonspecific. Taken together, our data support an important link between neurexin and presynaptic actin organization to mediate presynaptic assembly, stability, and function.

### Uncovering novel candidates for neurexin interaction

Several of the synaptic proteins enriched in our proteomic analysis have not been previously linked to neurexin signaling. DDI-1 has been implicated in negative regulation of synaptic assembly in *C*. *elegans*, with its mutants displaying a significant increase in synaptic density along the dorsal nerve cord [35]. The immunoglobulin cell adhesion molecule SAX-7 has been implicated in maintaining placement of neurons and their axons [36], and more recently genetically linked to RAB-3, suggesting a possible function in SV exocytosis [37]. None of the mutants we analyzed perfectly recapitulated the *nrx-1(-)* mutant phenotype, suggesting that neurexin may function as a signaling hub upstream of several different signaling pathways for synapse assembly, stability, and maturation. Altogether, our data suggests that neurexin may interact with several important structural, organizational, and functional synaptic players to mediate presynaptic development through distinct signaling pathways.

Interestingly, we also found hits in other classes of proteins, including those involved in the direct regulation of exocytosis (including SNARE proteins), in autophagy, in calcium signaling, as well as various kinases and axon guidance molecules. This suggests that there may be non-canonical functions of neurexin that together characterize its complex role in presynaptic regulation.

### Importance of negative control selection in proteomics analyses

An important contribution of this study is our in-depth analysis of several different conditions and negative control strains. To be useful, proteomic screens must have a good signal-to-noise ratio. This issue is emphasized in biotinylation experiments due to the presence of proteins with high endogenous biotin association such as carboxylase enzymes [34]. Our goal in comparing our experimental strain to 3 different negative control strains, including one generated specifically for this experiment, was to identify the comparison with the best ratio. We concluded that comparison to a wild-type strain (N2 Bristol) was too permissive, while comparison to an over-expressed cytosolic TurboID was too restrictive. Generation of a specific control strain in which TurboID was still tethered to neurexin and expressed at endogenous levels off the endogenous promoter, but in which neurexin’s clustering at the active zone was specifically abrogated, furnished us with the greatest enrichment of expected classes of proteins. Moreover, this control has the added advantage of presumably being trafficked through the secretory pathway in much the same way as the full-length tagged neurexin protein, thus removing from our analysis interactors outside the synapse. In fact, the localization specificity obtained from this control may be comparable to that obtained using newer methods such as split-TurboID or depletion of endogenously biotinylated carboxylases, without the need to limit biotinylation to a known neurexin-based protein complex or the use of specifically engineered strains and additional steps, respectively [34,38]. We conclude that selection of appropriate negative controls is a critical aspect of proteomic experiments and was instrumental in allowing us to identify novel interactors of neurexin’s ICD, including those involved in actin assembly.

### Limitations of the present study and future directions

The success of proteomics experiments is determined by the specificity and applicability of the controls to which experimental specimens are compared. In this study, we compared our experimental strain to multiple different control strains, and concluded that the specific deletion of neurexin’s localization motif (ΔPBM) was the most appropriate control because it disrupts neurexin’s clustering at active zones. However, this perturbation may also impact the total amount of NRX-1-TurboID protein at the cell surface, leading to an overall lower level of biotinylation. The identification of synapse-specific candidates and known neurexin-interactors mitigates this concern, suggesting that overall levels of TurboID expression may not be as critical as local enrichment.

Proximity ligation experiments do not necessarily indicate a direct interaction. It is possible that tagging the intracellular domain of neurexin will lead to the identification of all active zone proteins, given their close proximity to one another. Evidence of direct interaction is still required (as has been demonstrated previously for one of our top hits, SYD-1, in *Drosophila* [17]). Moreover, it can be argued that tagging any active zone protein would lead to the identification of actin-binding proteins, given the enrichment of actin within the presynaptic bouton. We believe this is unlikely because previous experiments (albeit conducted in different labs and thus under slightly different conditions) in which a core active zone protein was tagged with TurboID failed to identify an enrichment of actin-binding proteins.

This study provides a roadmap for future investigation of neurexin’s intracellular interactions. The candidates identified here must be further validated by demonstrating direct interaction, localization to presynaptic compartments, or common genetic pathways. Overall, the preponderance of actin-binding proteins suggest a mechanism by which neurexin may function in presynaptic assembly and maturation. Future studies must dissect out the precise molecular mechanisms by which this important and disease-relevant protein functions.

## Materials and methods

### Strains

Worms were grown at 23°C on nematode growth medium (NGM) plates seeded with *Escherichia coli* OP50 as a food source. Imaging analysis was performed at the larval L4 stage.

*C*. *elegans* strains used in this study can be found in S1 Table.

### Transgenic lines

Transgenic lines were prepared by gonadal microinjection of expression vectors for overexpression models or CRISPR/Cas9 for endogenous transgene expression or editing. Overexpression clones were made in the pSM vector [39]. Pan-neuronal overexpression was driven by the promoter rgef-1 and DA9-specific expression was driven by the mig-13 promoter. Standard techniques were used in the preparation of the plasmids and transgenic strains were prepared by microinjection using 1 to 5 ng/μl of plasmid DNA and coinjected with markers Podr-1::RFP at 100 ng/μl.

### Generation of neurexin TurboID-tagged by CRISPR/Cas9

Neurexin was TurboID-tagged by CRISPR-mediated insertion of TurboID into the endogenous neurexin genomic locus either just after the transmembrane domain (“post-TM”) or right before neurexin’s PDZ-binding motif (“pre-PBM”) near the C terminus of the protein. To create the “pre-PBM” neurexin-TurboID strain used for the proteomics experiments in this study, the microinjection mix contained a crRNA with a guide RNA chosen close to the site of interest (3′ AAACGGAAACGGGAATGGG 5′), Alt-R S.p. Cas9 Nuclease V3 (IDT, Cat. # 1081058) and a repair template generated by PCR that included the TurboID gene embedded with unc-119(+) cassette flanked by loxP sites within TurboID’s intron and a 96 bp and 97 bp homology arms to Cas9 cut site. DP38 [unc-119(ed3) III] strain was crossed with TV18675 (wyIs685 [Pmig-13::GFP::cla-1S + Pmig13::tdTomato::rab-3]) and the resulting strain PT23 [unc-119(ed3) III; nrx-1(kur2), wyIs685 V] was used for the injections. Transgenic animals were then selected based on behavioral rescue of the UNC phenotype by the expression of unc-119(+) and confirmed by PCR genotyping. Unc-119(+) cassette was then deleted by overexpression of Cre recombinase performed by microinjection of the plasmid pDD104 (Peft-3::Cre; Adgene). Genetic edited animals were selected based on UNC phenotype and confirmed again with PCR genotyping. Lastly, animals were out-crossed with N2 males to select away the unc-119(ed3) III allele resulting in the PTK31 [nrx-1(kur2), wyIs685 V] used for imaging and PTK57 [nrx-1(kur2) V] strain used for the proteomics in this study.

### Generation of neurexin-ΔPBM-TurboID by CRISPR/Cas9

Removal of the PBM from the neurexin-TurboID strain (PTK57) was performed by CRISPR-mediated deletion. For this purpose, a co-CRISPR methodology [40] was employed. PTK57 was injected with a mix containing crRNA targeting the PBM region (guide sequences used: 3′ TTTCTTCAATCAAAACTCAA 5′, 3′ AGAAAAAGGATTTTAAAGAG 5′ and 3′ GGTGGCACAGGAGGAACGGG 5′), a repair templated for the deletion with 100 bp homology arms flanking the PBM, as well as a crRNA targeting the dpy-10 gene and its repair template [40]. Roller worms were then singled and genotyped for PBM deletion, and these worms were subsequently passed to select away from the dpy-10 allele resulting in the PTK226 [nrx-1(kur43) V] strain used as a control in our proteomics experiments.

### Protein extraction for proteomics and western blotting

Protein extracts were prepared by harvesting synchronized worms enriched for adults with M9 onto a microcentrifuge tube followed by 3 M9 washes and 2 milli-q H_2_O washes. In the condition with added biotin, prior to the washes, worms were incubated at room temperature (22°C) in M9 buffer supplemented with 1 mM biotin, and *E*. *coli* OP50 for 2 h. After the washes, lysis buffer (150 mM NaCl; 50 mM Tris (pH 8) and 0.1% NP-40) was added to the samples which were snap frozen in liquid nitrogen. This was followed by 3 cycles of pestle grinding/snap freezing and lastly by a 20,000g centrifugation at 4°C for 20 min. The protein content on the extracts was quantified using Pierce BCA Protein Assay Kit (Cat. #23225). Three replicates were used for each of the 8 conditions (neurexin-TurboID, neurexin-ΔPBM-TurboID, neuronal cytosolic TurboID, and wild type/N2 each with and without added biotin).

### Western blotting

A total of 10 μg of protein extracts were separated by 10% SDS-PAGE Tris-glycine polyacrylamide gel electrophoresis, and 0.2 μm nitrocellulose membranes were used for the transfer in Towbin buffer for 4 h at constant 280 mA. Blots were incubated for 5 min with Ponceau S (0.1% (w/v) Ponceau S in 5% glacial acetic acid) for total protein visualization to control for possible loading differences. For immunodetection of biotinylated proteins, membranes were blocked in 7% milk in 1xTBS and 0.01% Tween-20 and streptavidin-HRP immunostaining (1:5,000, Invitrogen cat. #19534–050) was performed at room temperature for 1 h in blocking solution. After 3 washes with TBST, membranes were covered with SuperSignal West Femto Maximum Sensitive Substrate (Thermo Scientific, Cat. #34095) according to manufacturer’s instructions and chemiluminescence was then documented using Azure 600 Western Blot Imaging System (Azure Biosystems).

### Proteomics streptavidin pull-downs and mass spectrometry

A total of 100 μg of protein extracts were incubated with freshly washed Pierce Streptavidin Plus Ultra-Link Resin (Thermo Scientific, Cat. #53117) in protein binding buffer [150 mM NaCl; 50 mM Tris (pH 8); 10 μm ZnCl2; 0.5 mM DTT; 1:10 complete protease inhibitors (Sigma-Aldrich, Cat. #P2714); 10 mM sodium butyrate] for 6 h at 4°C in a rotation wheel. Supernatant was discarded and streptavidin beads were resuspended in 100 μl of protein binding/wash buffer (350 mM NaCl; 50 mM Tris (pH 8); 10 μm ZnCl2) followed by loading the samples into the desalting plate (Orochem OF1100 96-well plate) and 5 washes with protein binding/wash buffer. To reduce disulfide bonds, a 1-h incubation at room temperature with 100 μl of 5 mM of DTT in 50 mM ammonium bicarbonate was done, which was followed by blocking reduced cysteine residues with 20 mM of iodoacetamide (100 μl/well) during 30 min in the dark also at room temperature. After blocking, flow-through was discarded and trypsin incubation (250 ng/well) was performed overnight with a 60% ACN in 0.1% TFA (25 μl) wash right after. Desalting was then performed as previously described [41] followed by mass spectrometry. Briefly, samples were loaded onto a Dionex RSLC Ultimate 300 (Thermo Scientific), coupled online with an Orbitrap Fusion Lumos (Thermo Scientific). Chromatographic separation was performed with a two-column system, consisting of a C-18 trap cartridge (300 μm ID, 5 mm length) and a picofrit analytical column (75 μm ID, 25 cm length) packed in-house with reversed-phase Repro-Sil Pur C18-AQ 3 μm resin. Peptides were separated using a 60 min gradient from 4% to 30% buffer B (buffer A: 0.1% formic acid, buffer B: 80% acetonitrile + 0.1% formic acid) at a flow rate of 300 nl/min. The mass spectrometer was set to acquire spectra in a data-dependent acquisition (DDA) mode. Briefly, the full MS scan was set to 300 to 1,200 m/z in the orbitrap with a resolution of 120,000 (at 200 m/z) and an AGC target of 5x10e5. MS/MS was performed in the ion trap using the top speed mode (2 s), an AGC target of 1x10e4 and an HCD collision energy of 35. Raw files were searched using Proteome Discoverer software (v2.4, Thermo Scientific) using SEQUEST search engine and the SwissProt *C*. *elegans* database. The search for total proteome included variable modification of N-terminal acetylation and fixed modification of carbamidomethyl cysteine. Trypsin was specified as the digestive enzyme with up to 2 missed cleavages allowed. Mass tolerance was set to 10 pm for precursor ions and 0.2 Da for product ions. Peptide and protein false discovery rate was set to 1%. The dataset was then processed with logarithm transformation (to fit the data to a normal distribution, as proteomics data have a positively skewed distribution), normalized to total protein levels, and missing values were imputed (replaced using the Probabilistic Minimum Imputation for label-free data, as described in [42]). Mass spectrometry raw files are deposited in the repository Chorus under the project number 1794 and can be downloaded using the following link: https://chorusproject.org/anonymous/download/experiment/9f9c80cd0f9d454cafa3533b34e80c55.

### Immunohistochemistry

Immunohistochemistry was performed using the freeze-crack protocol described in www.wormbook.org with the following modifications. Ice cold 4% PFA was used as fixative solution with a 2-h incubation at 4°C. This was followed by blocking with 1% Triton X-100, 1 mM EDTA (pH 8), 0.1% BSA, and 7% normal donkey serum in 1× PBS for 4 h at room temperature. Incubation with mouse anti-BirA primary antibody (1:250, Abcam, Cat. #Ab232732) was performed overnight at 4°C. Secondary antibody incubation was also performed overnight at 4°C with donkey anti-mouse Alexa 647 (1:250, Invitrogen, Cat. # A-31571).

### Confocal microscopy

Imaging was performed at room temperature in live *C*. *elegans* grown at 23°C. An average of 20 mid-L4 stage hermaphrodite worms were paralyzed with 10 mM levamisole (Sigma-Aldrich) in M9 buffer and mounted on 5% agar pads for imaging. Animal stage was determined based on the correct stage of vulval development using DIC optics. Images of fluorescently tagged fusion proteins were captured with a Zeiss Axio Observer Z1 microscope with a Plan-Apochromat 63× or 40× 1.4NA objective and a Yokagawa spinning-disk unit attached to an EM-CCD camera.

### Image processing and data quantification

Using ImageJ (NIH), maximum-intensity projections were generated followed by cropping and straightening of the images. Puncta number was then quantified using a custom ROI-based MATLAB application using local mean thresholding and ROI watershed segmentation followed by parametric restriction to remove noise pixels [43]. Image levels, whenever required, were adjusted in Adobe Photoshop to show relevant features. In such cases, any images being compared were treated in the same manner.

### Enrichment analysis

An ANOVA test based on a generalized linear model was used in R to compare the experimental genotype (neurexin-TurboID) to the 3 controls in each condition. A log2 fold-change greater than 0 and a cutoff of 0.1 for overall *p*-value and 0.05 for the pairwise *p*-value was used to determine whether a protein was enriched.

### Volcano plots

Volcano and Semi-Volcano plots were constructed using a custom MATLAB script which plotted the pairwise *p*-value (-log10) from the ANOVA analysis against the median fold change (log2) [44].

### Ontology analysis

GO and tissue analysis was performed using the enriched (up-regulated) portion of the proteomics hits from the neurexin-TurboID strain samples when compared to the control samples. Figures in this manuscript focus on neurexin-TurboID versus neurexin-ΔPBM-TurboID enriched hits as described in the text. Both enrichment analyses were carried out using Wormbase’s Gene Enrichment Suite [45].

### Other plots

GraphPad Prism 9.0 (GraphPad Software, La Jolla, California, United States of America) software was used to generate the plots for Figs 2, 3, and 4. An ANOVA test was used to test for significance compared to controls and all data are represented as mean ± SEM, and significance is defined as *p < 0.05, **p < 0.01, or ***p < 0.001, unless otherwise noted.

### Clustering analysis

PCA analysis, k-means clustering analysis, and related graphs were done using the plotly, stats, and factoextra R packages [46–48]. The *prcomp* function was used to generate the principal components from a transposed matrix of the preprocessed proteomics data (S2 Table) using a maximal rank value of 4, then the 2 components accounting for the highest variance were used as axes for the PCA plot. The optimal k-means cluster analysis was done using the *fviz_nbclust* function and the clusters were plotted using *eclust* function, with the number of clusters set based on the optimal number of clusters.

## Supporting information

S1 Fig Assessment of control strains. (A) Western blot of biotin-tagged proteins of our experimental strain (neurexin-TurboID, right columns) compared to 2 controls (wild type, left columns and neurexin-ΔPBM-TurboID, middle columns) in the basal, non-enriched biotin condition. Total protein levels (as assessed by Ponceau staining, lower blot) were used as a loading control. (B) Volcano plot of genes corresponding to the proteins enriched in our experimental strain (neurexin-TurboID) compared to control (neurexin-ΔPBM-TurboID), in either the basal (left) or enriched (right) biotin conditions. (C) Tissue Enrichment Analysis for the proteins enriched in neurexin-TurboID relative to the neurexin-ΔPBM-TurboID control in the enriched biotin condition. (D) Overlap between the hits in C with hits from ELKS-1-TurboID from Artan and colleagues [34], highlighting the subset of actin-related genes.
(TIF)

S2 Fig Principal component analysis and clustering of proteomics samples. (A) Representation of the proteomics samples using the 2 principal components that account for the highest variance. (B) Determination of the optimal number of clusters using kmeans clustering. (C) Kmeans clustering of proteomics samples using the optimal number of clusters (4) and Euclidean distance.
(TIF)

S3 Fig Neurexin RAB-3 phenotype present in some candidate interactors. (A) Schematic of the worm tail showing the region of the images. (B) Images of the DA9 motor neuron showing RAB-3-TdTomato fluorescence, which is normally restricted to the synaptic region in control (CRTL) and neurexinTurboID worms but reveals small asynaptic puncta in *nrx-1(-)* mutants, neurexin-ΔPBM-TurboID worms, and *frm-4(-)* mutants (second row). Arrowheads display examples of asynaptic RAB-3 puncta not present in wild type. Scale bars: 10 μm.
(TIF)

S4 Fig GO analysis connectome showing prominent enrichment of actin-related proteins. Connectome displaying the different GO terms found to be enriched in the samples. Actin-related terms are highlighted by the dotted red segment ROI of the map.
(TIF)

S1 Raw Images Raw images of western blots. Raw images of blots included in Figs 2B and S1A.
(PDF)

S1 Table Strain list. (XLSX)

S2 Table Processed Proteomics Data. Spreadsheet containing the entire preprocessed proteomics dataset, Biotin- and Biotin+ subsets, and list of up-regulated genes in the Biotin- and Biotin+ subsets.
(XLSX)

S3 Table ROI analysis. Puncta count and puncta size data used for graphs in Figs 1E, 4C, and 4F.
(PZFX)

## Abbreviations

AZ: active zone
DDA: data-dependent acquisition
GO: Gene Ontology
GS1: Gelsolin segment 1
ICD: intracellular domain
NGM: nematode growth medium
PBM: PDZ-binding motif
sCAMS: synaptic cell-adhesion molecules
SV: synaptic vesicle
TEA: tissue enrichment analysis
