# Supplementary figures and images for: Characterization of the intracellular neurexin interactome by in vivo proximity ligation suggests its involvement in presynaptic actin assembly
Source: PLoS Biol. 2024 Jan 22;22(1):e3002466. doi: 10.1371/journal.pbio.3002466 (PMC10802952; doi:10.1371/journal.pbio.3002466)

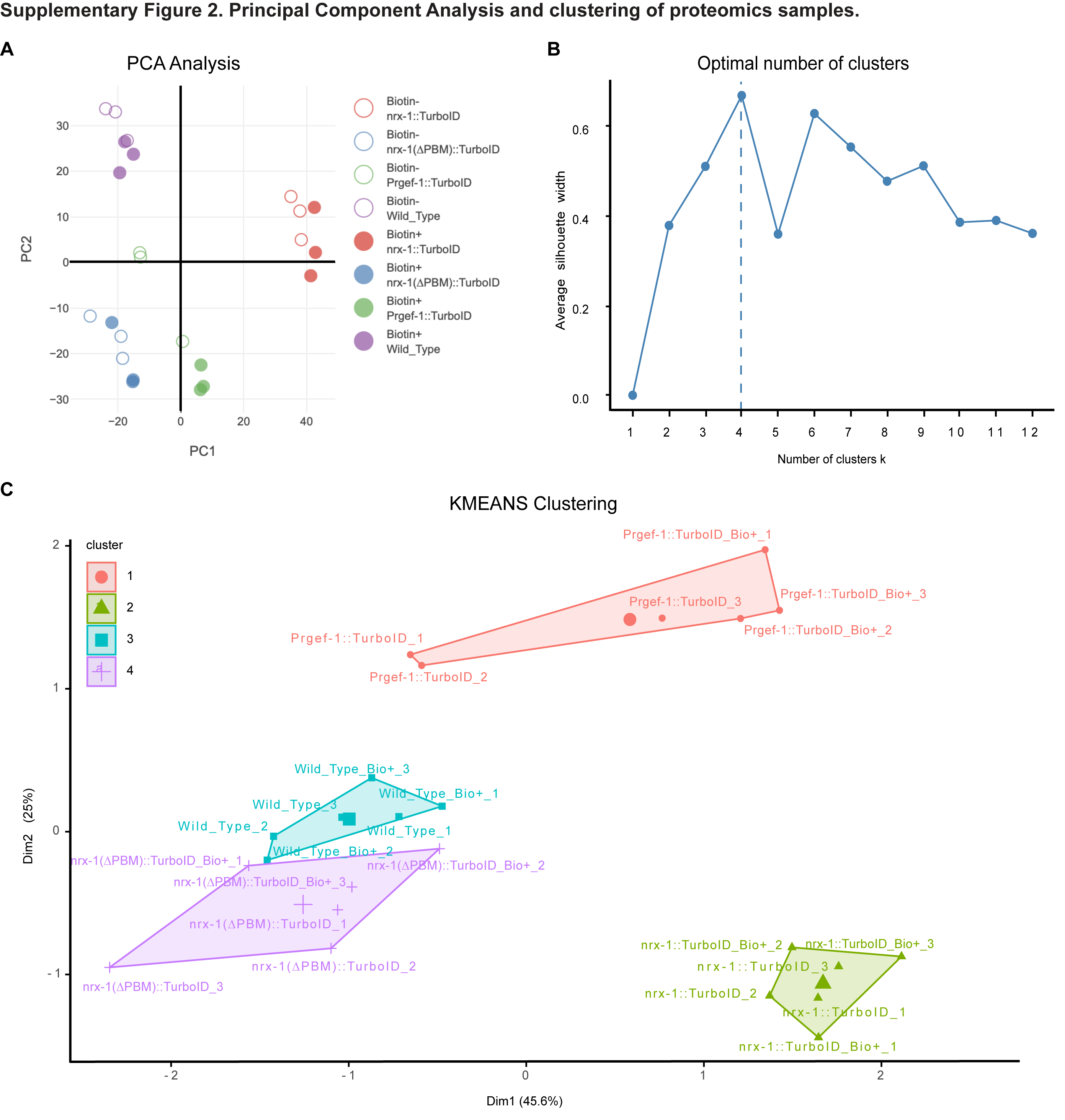

Supplement: S2 Fig — (A) Representation of the proteomics samples using the 2 principal components that account for the highest variance. (B) Determination of the optimal number of clusters using kmeans clustering. (C) Kmeans clustering of proteomics samples using the optimal number of clusters (4) and Euclidean distance. (TIF) [file pbio.3002466.s002.tif]

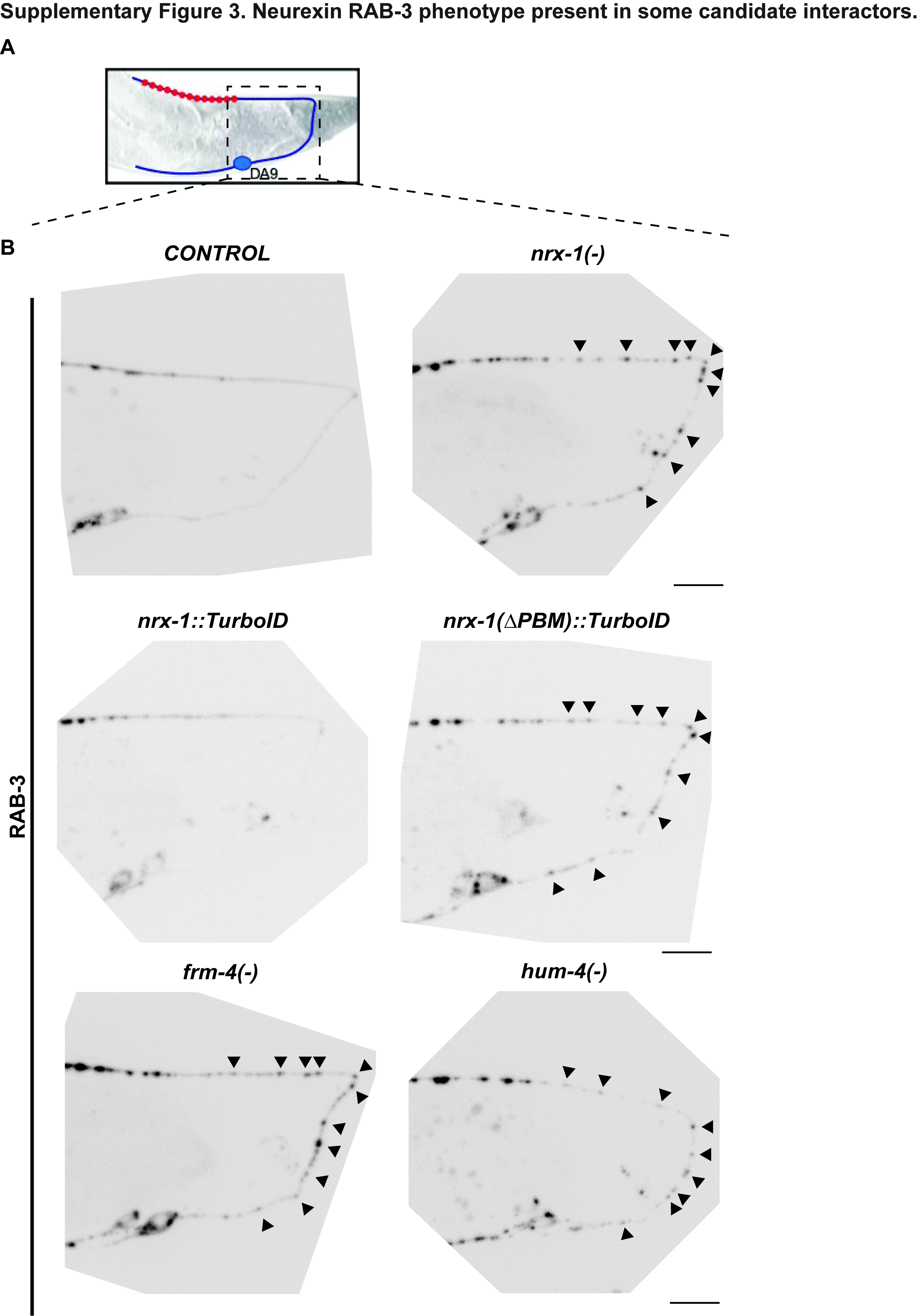

Supplement: S3 Fig — (A) Schematic of the worm tail showing the region of the images. (B) Images of the DA9 motor neuron showing RAB-3-TdTomato fluorescence, which is normally restricted to the synaptic region in control (CRTL) and neurexinTurboID worms but reveals small asynaptic puncta in nrx-1(-) mutants, neurexin-ΔPBM-TurboID worms, and frm-4(-) mutants (second row). Arrowheads display examples of asynaptic RAB-3 puncta not present in wild type. Scale bars: 10 μm. (TIF) [file pbio.3002466.s003.tif]

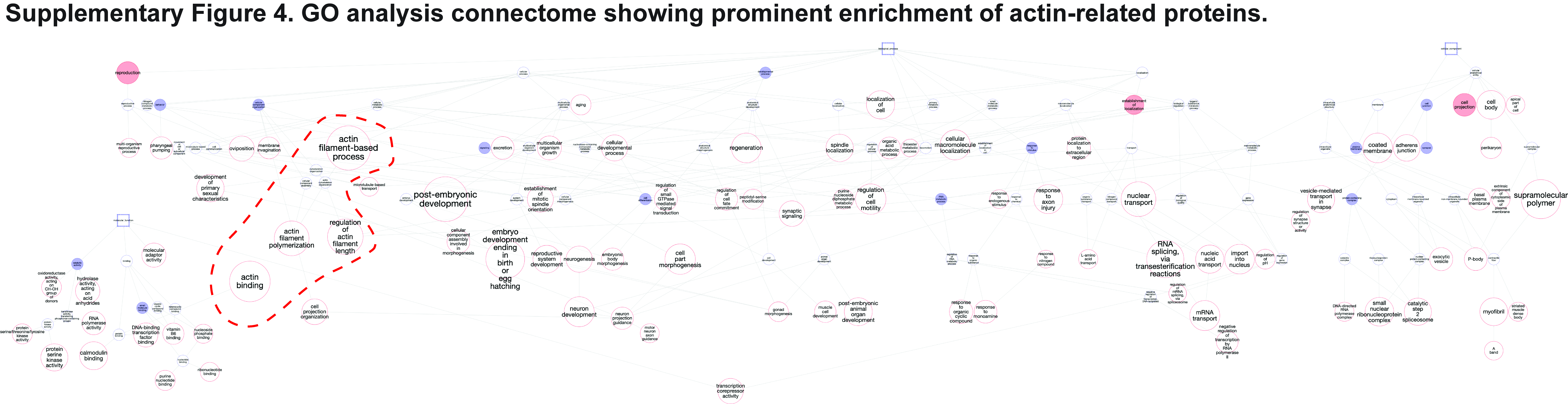

Supplement: S4 Fig — Connectome displaying the different GO terms found to be enriched in the samples. Actin-related terms are highlighted by the dotted red segment ROI of the map. (TIF) [file pbio.3002466.s004.tif]

Ponceau S

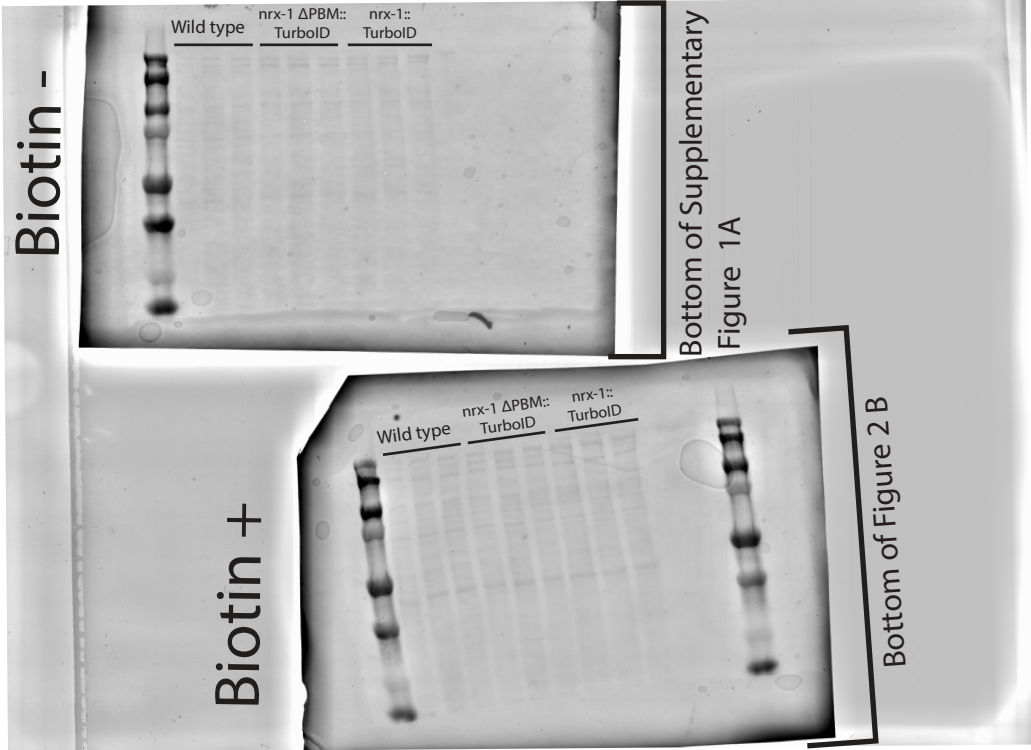

Streptavidin-HRP

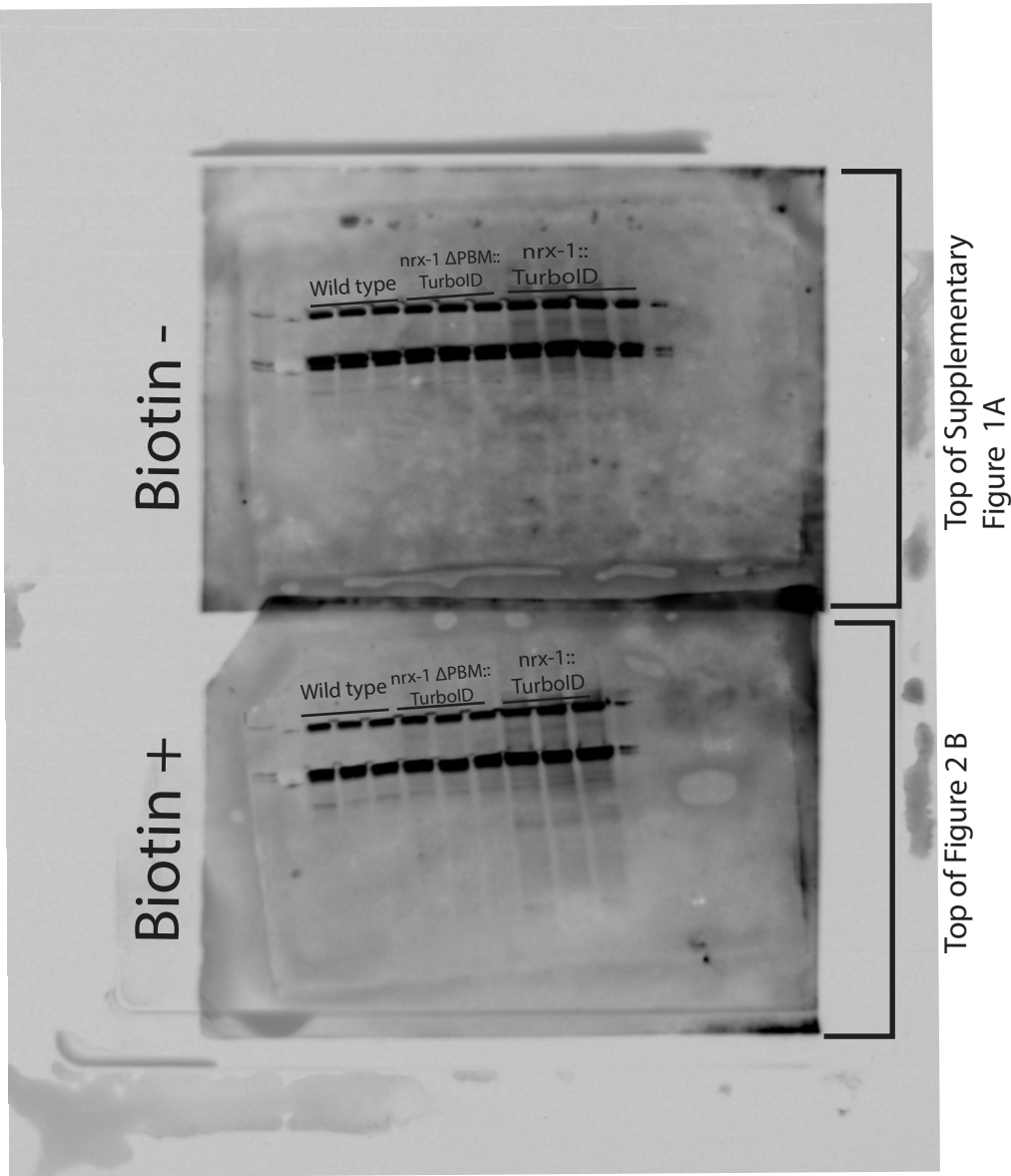

Supplement: S1 Raw Images — Raw images of blots included in Figs 2B and S1A. (PDF) [file pbio.3002466.s005.pdf]
